# Patient, thyroid, and surgeon related factors that make thyroidectomy difficult-cohort study

**DOI:** 10.1016/j.amsu.2019.11.010

**Published:** 2019-11-23

**Authors:** Sapana Bothra, Mayilvaganan Sabaretnam, Asish Kannujia, Gyan Chand, Gaurav Agarwal, S.K. Mishra, Amit Agarwal

**Affiliations:** a Department of Endocrine Surgery, India; b Department of Anaesthesia, Sanjay Gandhi Postgraduate Institute of Medical Sciences, Rae Bareilly Road, Lucknow, 226 014, India

**Keywords:** Thyroidectomy, Heart rate, Surgeon

## Abstract

**Introduction:**

When thyroidectomy is performed under optimal conditions within a milieu of sound anatomical and physiological knowledge combined with meticulous surgical skills, complications are minimal. However, thyroidectomy can be difficult, and its complications can be life-threatening. The factors that predict difficult thyroidectomy can be patient-, thyroid-, or surgeon-related, and we aimed to study these three factors. .

**Materials and methods:**

This prospective study was performed in a tertiary care center between September 2016 and March 2017. We developed and validated modified thyroidectomy difficulty scale (TDS), with 11 items. Preoperatively, height, weight, neck length, and other parameters were recorded. Postoperatively, the modified TDS form was filled out by the surgeon and assistant, blinded to each other's responses. The minimum score was 19 and maximum was 54. The surgeon's baseline pulse rate was monitored throughout the procedure using a pulse oximeter probe that was On-The-Go (OTG) compatible. The probe was placed over the ear lobule/pinna of the surgeon and connected to an Android phone that was comfortably placed in the surgeon's pocket inside the gown. An application USB SPO_2_, was used in recording the pulse rate.

**Results:**

A total of 52 patients undergoing hemi- or total thyroidectomy were included in this study. All had benign cytology on fine needle aspiration cytology (colloid, 71.42%). A total of 104 modified TDS questionnaires filled by the operating surgeon and assistant were analyzed. The pulse rate of the operating surgeon, as measured by the novel pulse oximeter, was recorded in 52 surgeries. The minimum score was 20, maximum score was 35.50, and mean score was 26.85 ± 2.80. There was an interobserver agreement in most domains of the modified TDS except mobility. The surgeon was found to have the maximum heart rate when performing recurrent laryngeal nerve (RLN) dissection in 38 patients (73.07%).

**Discussion:**

We found that majority of the trainees found thyroidectomy to be a vigorously intense activity. Thyroidectomy is a demanding surgery, which requires meticulous identification and dissection of the RLN and parathyroid glands for optimum outcome.

## Highlights

• Thyroidectomy performed under optimal conditions with sound anatomical knowledge can have minimal complications.
• Thyroidectomy can be difficult.
• Majority of the trainees found thyroidectomy to be a vigorously intense activity.

## 1 Introduction

During the early part of the twentieth century, thyroidectomy was associated with high mortality and morbidity rates, and thyroid surgeries were considered barbaric and banned by the French Medical Society because of the associated mortality [1]. With improved understanding of thyroid function, thyroid surgeries are much safer than they were before; however, similar to any other surgical procedure, thyroidectomy is fraught with complications. When thyroidectomy is performed under optimal conditions, within a milieu of sound anatomic and physiologic knowledge combined with meticulous surgical skills, the incidence of complications is minimal [2]. The mindset involved in the operation is best modified to understand the proposition that if the thyroid gland is carefully devascularized, it can be totally separated from the surrounding vital structures [2].

Thyroidectomy, a simple surgery, can be complicated by a myriad of factors. In the present scenario, thyroidectomy has virtually zero mortality and extremely low morbidity rate when performed by high-volume, trained endocrine surgeons [[3], [4], [5]]. However, thyroidectomy can be difficult, and its complications can be life-altering and rarely life-threatening. The factors that predict difficult thyroidectomy can be patient-, thyroid-, or surgeon-related. The surgeon performing thyroidectomy should have proper training, and the complication rate should be acceptable.

Thyroidectomy is a procedure that demands accurate identification of the nerves and parathyroid glands, and precise dissection to preserve the blood supply for optimal outcomes and to avoid complications. The thyroid gland when enlarged and pathological can distort the normal anatomy with displacement of structures. Moreover, when the vascularity increases, as in toxic goiters and malignancy, it can result in difficult thyroidectomy. Patient-related factors such as obesity, short neck, cervical spondylitis, and other factors including the decision making for surgery can result in difficulty for the surgeon. The surgeon performing the thyroidectomy is affected by several factors such as peer pressure to perform, professional relationship with the consultant, marital issues, basic surgical training, years and place of training, type of personality, personal health habits and health issues, and also the mentor and project/thesis (which is mandatory for completion of the fellowship/superspecialty training), with the burnout rate ranging from 30 to 38% [[6], [7], [8], [9], [10]]. The consultant surgeon in this medicolegal era must balance between surgical training and patient safety. Complication rates, blood loss, and operative time serve as surrogates for difficulty. Difficulty scales have been developed for other procedures, often as a means to quantify the learning curve [11]. The notion of difficulty of thyroid surgery in literature remains subjective and is limited to case reports, opinions, and technique papers [12,13]. Currently, there are no measures of difficulties in thyroidectomy as there are for other operations. Thyroidectomy requires a more objective measurement of difficulty and evidence-based identification of patient-, disease-, and surgeon-related factors. A novel 4-item 20-point TDS was developed by David F. Schneider [14]. In this study, we aimed to analyze the factors related to the thyroid gland, patient, and surgeon in patients undergoing thyroidectomy in a tertiary referral institution and how these factors can make the thyroidectomy difficult.The results can make future readers and surgeons to be cautious when encountered with these difficult factors when performing safe thyroid surgery.

## 2 Materials and Methods

**Fig. 1:**
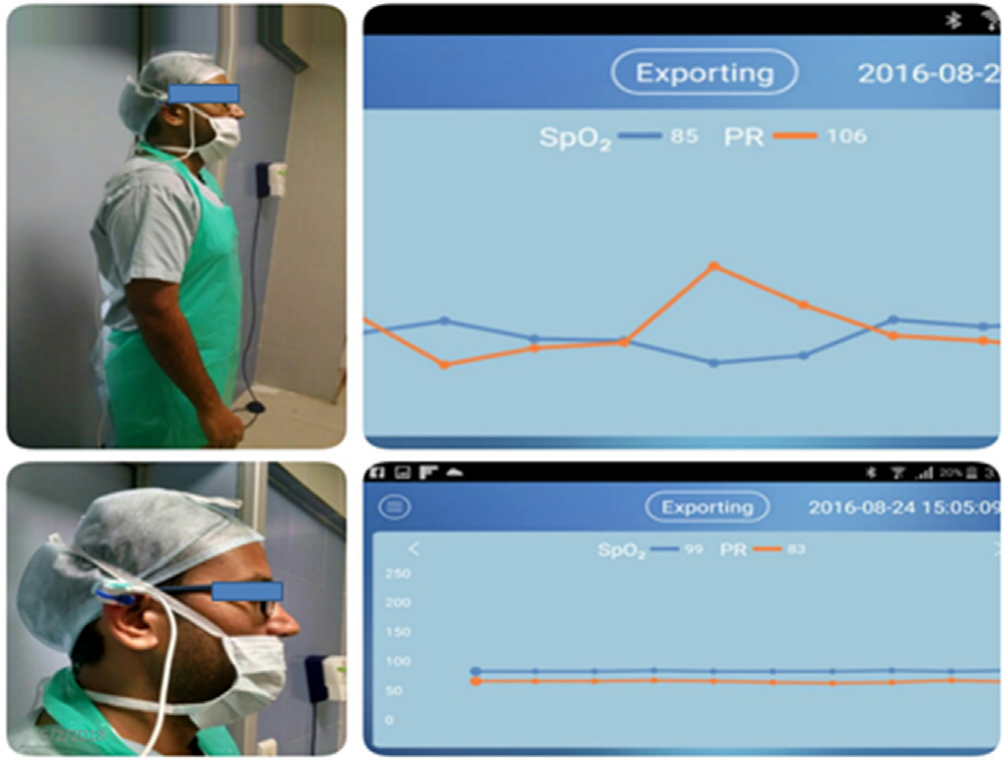
Placement of the OTG-compatible pulse oximeter probe over the pinna and two recordings of SPO2 and pulse rate of the surgeon in two different procedures.

This prospective study included 52 patients undergoing hemi- or total thyroidectomy for cytologically proven benign thyroid disorders.The patients who consented to be the part of the study were included in this study and patients with malignant cytology and not consenting were excluded. We used a validated modified difficulty thyroidectomy scale (TDS) (the minimum score was 19 and maximum was 54), with 11 items, which was filled by the surgeon and first assistant, immediately after the surgical procedure. The various patient-related parameters including demographic profile, height, weight, neck length, hormonal profile, fine needle aspiration cytology (FNAC), and ultrasound (US) findings, and other parameters were recorded in the forms. An On-The-Go (OTG)-compatible SPO_2_ probe was placed over the ear lobule/pinna of the surgeon, and connected to an Android phone that was comfortably placed in the surgeon's pocket inside the gown. An application USB SPO_2_, marketed by Berry, was used to record the pulse rate. The operating surgeon's baseline pulse rate was recorded before scrubbing, and the probe recorded the SPO_2_ and pulse rate throughout the surgery in the Android phone (Fig. 1). The recordings were evaluated by the two first authors, who were the primary investigators of this study. The point of maximum heart rate elevation was analyzed based on the time of dissection and the data recorded on the Android application. The surgeons were all physically fit, and not on any drugs. The operating theater atmosphere, including the scrub team did not change, and the setting remained the same for the six surgical consultants and trainees who performed this study.When the surgery was done by a trainee a consultant scrubbed and assisted the procedure. There was no bullying or ragging during any of these surgical procedures.

The modified Karvonen formula was used to calculate the target heart rate (220-age) and heart rate reserve (HRR = target heart rate–resting heart rate). Using this HRR, we classified the surgical procedure as light-intensity activity with 30–40% of the HRR, moderate-intensity activity with 40–60% of HRR, and vigorous-intensity activity with 60–90% of HRR [15]. This work has been reported in line with the STROCSS criteria [16].

## 3 Modified TDS

This modified TDS includes 11 different items that can contribute to difficulty in thyroidectomy, including vascularity of the gland, friability, mobility/fibrosis, gland size, tubercle of Zuckerkandl (TZ) (grade), recurrent laryngeal nerve (RLN) (type, relation to TZ, relation to ligament of Berry, vascularity of the ligament, relation to inferior thyroid artery (ITA), and branching and its trajectory), parathyroid gland (identification, location, and autotransplantation), retrosternal extension, external branch of the superior laryngeal nerve (EBSLN) (type), neck contour, and thyrothymic remnant.This modified TDS scale was validated in iodine deficient population by a pilot study [17].

## 4 Statistical analysis

Descriptive statistics of the continuous data were presented using mean ± standard deviation or median as appropriate. To compare the means between two or three groups, independent samples *t*-test or one-way ANOVA test was used. To compare the median between two or three or more groups, Mann Whitney *U* test or Kruskal Wallis H test was used. A P-value<0.05 was considered statistically significant. Statistical analysis was done using Statistical Package for the Social Sciences version 23 (SPSS-23, IBM, Chicago, USA).

## 5 Results

A total of 52 patients underwent hemithyroidectomy (24 patients, 46.15%) or total thyroidectomy (28 patients, 53.84%) for benign etiology. Of these patients, 44 (84.61%) were female and 8 (15.38%) were male. Forty-one patients (78.84%) were euthyroid and 11 (21.15%) were hyperthyroid. The FNAC was colloid in 71.42%. The pulse rate of the surgeon was recorded in all 52 surgeries and analyzed. A total of 104 responses of the modified TDS filled by the operating surgeon and assistant were analyzed. The minimum score was 20, maximum score was 35.50, and mean score was 26.85 ± 2.80. There was interobserver agreement in most domains except mobility. The heart rate was observed to be maximum during RLN dissection in 38 patients (73.07%), followed by superior pole ligation in 10 (19.23%) and parathyroid dissection and middle thyroid vein ligation in 2 patients each. The incision used was a 6-cm skin-crease in 40 patients (76.92%). The correlation between patient-, thyroid-, and surgeon-related factors with the modified TDS is provided in Table 1, Table 2, Table 3, respectively.

**Table 1:** Distribution of TDS score according to the patient-related factors.

Variable's	Mean ± SD	Mean ± SD	P value
Age (<40, >40), years	26.71 ± 2.62	27.03 ± 3.30	0.709
Sex (male, female)	28.88 ± 1.26	26.49 ± 2.90	0.027
Weight (<50, >50), kg	26.27 ± 3.17	26.96 ± 2.75	0.461
Height (<150, >150), cm	26.10 ± 1.63	26.85 ± 2.96	0.581
Neck length (adequate, short)	26.76 ± 2.89	28.50 ± 0.50	0.305
Comorbidity (present, absent)	26.67 ± 2.63	29.00 ± 4.60	0.116
Thyroid status (euthyroid, hyperthyroid)	26.50 ± 2.90	29.00 ± 3.50	0.009

Independent samples *t*-test used**,** p < 0.05 significant.

**Table 2:** Distribution of TDS score according to thyroid-related factors.

Surgeon Factors Mean (TDS Score)	TDS score (median)					p-value
Thyroid gland factors score	1	2	3	4	5	
Vascularity	25.25	25.00	28.50	29.50	34.00	0.001
Friability	23.50	26.00	29.25	29.50	35.50	0.001
Mobility	23.25	26.00	28.00	28.50	33.25	0.000
Gland size	24.00	26.00	26.00	28.50	32.50	0.004
Tubercle of Zukerkandl	21.58	29.38	35.28	–	–	0.126
#RLN type	26.50	29.00				0.969
#RLN relation to TZ	26.70	25.50				0.505
#RLN relation to the ligament of Berry	26.50	27.00				0.659
#Vascularity of the ligament of Berry	26.00	28.25				0.018
#RLN location in relation to ITA	26.25	28.00				0.593
#RLN branching	26.50	27.50				0.356
RLN anatomical course	26.50					–
#Parathyroid identification	26.50	26.00				0.505
Parathyroid location	26.50					–
#Parathyroid autotransplantation	26.50	26.00				0.928
#Retrosternal extension	26.50	26.50				0.899
EBSLN	26.50	26.75	30.25			0.321
#Neck contour	26.50	25.50				0.505
#Thyrothymic remnant	26.50	27.50				0.969

Kruskal Wallis H test used**,** #Mann Whitney *U* test, p < 0.05 significant.

**Table 3:** Distribution of TDS score according to surgeon-related factors.

Surgeon Factors Surgeon-related factors Score)	TDS score (mean ± standard deviation)		p-value
Years of experience (≤3-year trainee, >3 year consultant)	26.87 ± 2.57	26.84 ± 3.20	0.975
Basal HR (<80, >80)	26.60 ± 2.48	27.28 ± 3.38	0.407
Maximum HR (<120, >120)	26.82 ± 2.79	26.87 ± 2.91	0.947
Strap muscle (retracted, cut)	26.60 ± 2.46	27.77 ± 3.91	0.367
Number of assistant (1, >1)	28.50 ± 3.8	26.64 ± 4.7	0.584
Scrub nurse experience (<100, >100), cases	26.91 ± 3.22	26.78 ± 2.30	0.870
Operative time (2, >2), h	26.78 ± 2.93	27.00 ± 2.68	0.801
Adjuncts used (IONM) (yes/no)	27.27 ± 3.07	26.94 ± 2.25	0.717

Independent samples *t*-test used**,** p < 0.05 significant.

Among patient-related factors, age (<40 and >40 years), sex (male or female), weight (<50 or >50 kg), neck length (adequate or short), comorbidities (cardiac disease, obesity, hypertension, chronic pulmonary disease, diabetes, and renal failure [present or absent]), and thyroid hormonal status (euthyroid or hyperthyroid) were correlated with modified TDS score. There was a significant difference (p = 0.009) only in the mean TDS score of patients with hyperthyroidism when compared to that of their euthyroid counterparts.

Among thyroid-related factors, every parameter in the modified TDS was correlated with the median modified TDS score. The parameters of vascularity (p = 0.001), friability (p = 0.001), mobility (p = 0.000), gland size (p = 0.004), and vascularity of the ligament of Berry (p = 0.018) were significantly correlated.

Among surgeon-related factors, the years of surgical experience (<3-year trainees or >3-year consultant), basal heart rate (<80 or >80 per minute), maximum heart rate during the surgical procedure (<120 or >120 per minute), whether the surgeon divided or retracted the strap muscles, number of assistant surgeons (1, >1), scrub nurse experience (<100 or >100 thyroidectomies), operative time (<2 h or >2 h), and use of adjunct intraoperative nerve monitoring (IONM) did not correlate with the mean modified TDS scores.

The activity during surgery was of light intensity in 15.4% (n = 8), moderate intensity in 30.8% (n = 16), and vigorous intensity in 53.8% (n = 28). The various intensity levels (Table 4) did not significantly correlate with the modified TDS score (p = 0.450). There was no intraoperative or postoperative complication. Six patients required intravenous calcium infusion, but none had permanent hypoparathyroidism.

**Table 4:** Distribution of TDS score as per intensity level.

Intensity	N	Mean	Standard Deviation	Minimum	Maximum
Light	8	26.06	2.15	23.50	29.50
Moderate	16	27.03	2.99	23.00	35.50
Vigorous	28	26.99	2.85	20.00	32.50
Total	52	26.86	2.83	20.00	35.50

One-way ANOVA test: p = 0.424 (not significant).

## 6 Discussion

We found that a majority of the surgeons especially trainees (Table 4) found that thyroidectomy was a vigorous-intensity activity, and maximum pulse rate increase was noted during dissection near the RLN. Some surgeons had maximal heart rate elevation during dissection of the parathyroid gland, especially the middle thyroid vein ligation or superior thyroid artery ligation. The maximum pulse rate recorded in all procedure was 164/min, and this elevation lasted for 6 min when a third-year trainee tried to control the bleeding and managed it from the superior thyroid artery in a patient with Grave's disease undergoing total thyroidectomy.

The operating theater is a demanding environment where the surgeons, especially trainees, spend one-third of their time working. Key stress factors in the operating theater include operating with peers, bleeding, and distractions, time pressure, equipment problem, and interpersonal relationship with the anesthesiologist and nursing staff. Heart rate variability was shown to be a useful tool in revealing adverse effects of lifestyle and psychosocial stressors on the cardiovascular system [18]. The operating surgeon and assistant interpreted the continuous recording of this pulse rate to the events during the surgery, and this was recorded in the form. We have maintained a similar setting for all surgeries to exclude extraneous factors affecting the surgeons. The operating surgeon and assistant were not on any medications, and did not have any major personal or professional conflict on the day of surgery. One consultant who was on medication and a trainee on treatment for migraine were excluded from the study.

Among patient-related factors, most surgeons found it easy to perform surgery on female patients when compared to male patients. This might be due to the muscular nature of Indian men when compared to that of their female counterparts. The surgeons found that it was easier to operate on euthyroid goiters than on hyperthyroid goiters. Hyperthyroid goiters include Grave's disease, toxic multinodular goiter, and autonomous functioning thyroid nodule (AFTN). The increased vascularity and friability which are features of Graves's disease and also thyroiditis may be the reason for this difficulty.This finding of difficulty encountered in operating hyperthyroid patients was reported by Mok et al. in their study [19].

The thyroid-related factors that significantly correlated with the TDS score include vascularity, friability, mobility, gland size, and RLN vascularity. The vasa nervorum of the RLN, which imparts the name toothpaste sign, can result in difficult dissection of the RLN. The Nerve dissection can be made more difficult by the presence of tubercle of Zuckerkandl and if it is grade II or grade III then dissection can be difficult for even experienced surgeons.In relation to the thyroid-related factors, the assistant and surgeon scores were comparable in most domains, except for the mobility of the gland; because the assistant felt that the gland was more mobile.

Among surgeon-related factors, although the factors did not correlate significantly, the mean modified TDS score of the consultant and trainees did not significantly differ. This might be due to the bias of the consultant scrubbing for a difficult procedure, which is not always the case, and being a tertiary referral center catering to iodine-deficient population with large and long-standing goiters, which can result in difficult dissection even to the experienced consultant.The Trainees are given graded training and this can result in confident performance of thyroidectomies and might be the reason for scores not being significantly different.

A confident surgeon with a good understanding of the neck anatomy, the support of the consultant, and the desire to dissect meticulously near the nerve and parathyroid gland can provide good outcomes. The stress levels can be high if the operating surgeon is a trainee/resident. Further, the stress of residency can itself affect the performance during the surgery. A previous study addressed the surgeon's stress during colorectal surgery [20], but there is no study till date for thyroid surgery. We have tried to address this issue of stress during thyroidectomy by measuring the pulse rate of the endocrine surgeons, and used the intensity of activity as a surrogate marker of the stress. The patient-, thyroid-, and surgeon-related factors can independently make the thyroidectomy difficult. In this study, we included both hemi- and total thyroidectomies for benign thyroid disorders, and this was a limitation of our study.

## 7 Conclusion

Thyroidectomy is a demanding surgery and requires fine dissection and preservation of the vital structures such as the RLN and parathyroid glands. For trainees, it is a vigorous-intensity activity, especially during a certain part of the procedure, and imparts considerable stress. Graded training under mentorship of Trained faculty with adequate surgical exposure can make young surgeon's cope up with this stress and achieve optimum results.

## Ethical approval

Institute ethics committee no PGI/BE/549/2016 Dated 26.09.201.

## Sources of funding

No.

## Author contribution

Authors’ contribution: S.M. and SB contributed to the conception and design of the study. S.M., SB., and AK did the acquisition of data. S.M. and SB. did the analysis and interpretation of data. S.M. drafted the article. All authors SB SM AK GC AM GA SKM AA revised the article critically for important intellectual content and also the final approval of the version to be submitted.

## Conflicts of interest

No.

## Research registration number

Study ID ISRCTN72998252.

ISRCTN72998252 https://doi.org/10.1186/ISRCTN72998252.

ISRCTN72998252 https://doi.org/10.1186/ISRCTN72998252.

What makes thyroidectomy difficult: the thyroid, the patient or the surgeon?

## Guarantor

Dr.Mayilvaganan Sabaretnam.

## Provenance and peer review

Not commissioned, externally peer reviewed.
